# Lumbar Discectomy With Barricaid Device Implantation in Patients at High Risk of Reherniation: Initial Results From a Postmarket Study

**DOI:** 10.7759/cureus.20274

**Published:** 2021-12-08

**Authors:** Pierce Nunley, K Brandon Strenge, Kade Huntsman, Hyun Bae, Christian DiPaola, Allen R T, Andrew Shaw, Rick C Sasso, Ali Araghi, Blake Staub, Selby Chen, Larry E Miller, Michael Musacchio

**Affiliations:** 1 Orthopaedics, Spine Institute of Louisiana, Shreveport, USA; 2 Spine Surgery, The Orthopaedic Institute of Western Kentucky, Paducah, USA; 3 Spinal Surgery, Salt Lake Orthopaedic Clinic, Salt Lake City, USA; 4 Orthopedic Surgery, Cedar-Sinai, Santa Monica, USA; 5 Orthopaedics, UMass Memorial Healthcare, Worcester, USA; 6 Orthopaedics, University of California San Diego Health System, San Diego, USA; 7 Neurological Surgery, Lyerly Neurosurgery, Jacksonville, USA; 8 Orthopaedic Surgery, Indiana Spine Center, Carmel, USA; 9 Spine Surgery, The Core Institute, Sun City West, USA; 10 Orthopaedics, Texas Back Institute, Plano, USA; 11 Neurosurgery, Mayo Clinic, Jacksonville, USA; 12 Clinical Research, Miller Scientific, Johnson City, USA; 13 Orthopaedics, North Shore University Health System, Evanston, USA

**Keywords:** annular defect, sciatica, lumbar herniation, lumbar discectomy, barricaid

## Abstract

Background

Patients with large defects in the annulus fibrosus following lumbar discectomy have high rates of symptomatic reherniation and reoperation. An FDA randomized controlled trial (RCT) with a bone-anchored device (Barricaid, Intrinsic Therapeutics, Woburn, MA) that occludes the annular defect reported significantly lower risk of symptomatic reherniation and reoperation compared to patients receiving discectomy only. However, results of the Barricaid device in real-world use remain limited.

Methods

This was a post-market study to determine the real-world outcomes of the Barricaid device when used in addition to primary lumbar discectomy in patients with large annular defects. Main outcomes included leg pain severity, Oswestry Disability Index (ODI), adverse events, symptomatic reherniation, and reoperation. Imaging studies were read by an independent imaging core laboratory. This paper reports the initial three-month primary endpoint results from the trial; one-year patient follow-up is ongoing.

Results

Among 55 patients (mean age 41±13 years, 60% male), the mean percent reduction in leg pain severity was 92%, and the mean percent reduction in ODI score was 79%. The three-month rate of symptomatic reherniation was 3.6% and the rate of reoperation was 1.8%. The serious adverse event rate was 5.5%; no device migrations or fractures were observed.

Conclusion

Among patients with large annular defects following lumbar discectomy treated with the Barricaid device in real-world conditions, early results demonstrated clinically meaningful improvements in patient symptoms and low rates of symptomatic reherniation, reoperation, and complications, which were comparable to those observed with the device in an FDA-regulated trial.

## Introduction

Lumbar discectomy for the surgical treatment of disc herniation is an effective procedure for patients with persistent sciatica nonresponsive to conservative care. Surgical excision of the herniated nuclear material provides neural decompression and relief from sciatica symptoms. However, the defect in the annulus pulposus is typically left unrepaired and this weakened area serves as a potential location for disc reherniation. Post-discectomy reherniation rates are relatively low in the general population, ranging from 7% to 18% of patients within two years [1-4]. However, patients with a large defect in the annulus fibrosis have a more than two-fold greater risk for recurrence compared to those with small defects [5]. This population of patients at high-risk for recurrence forms the basis for the current study. A bone-anchored device (Barricaid, Intrinsic Therapeutics, Woburn, MA) may be implanted following completion of a limited discectomy procedure to occlude a large annular defect and reduce the risk for reherniation. A large FDA-regulated randomized controlled trial (RCT) confirmed the safety and efficacy of this treatment strategy [6]. However, results of the Barricaid device in real-world conditions remains limited. The primary purpose of this post-market study was to report early (three-month) outcomes of Barricaid device implantation when used in addition to a primary limited lumbar discectomy procedure in a patient population at high risk for reherniation. A secondary purpose of the study was to descriptively compare these early real-world outcomes to those from an FDA-regulated RCT.

## Materials and methods

Study design

This prospective, multicenter, single-arm postmarket study enrolled patients at high-risk for lumbar disc reherniation based on large annular defect measurements who were treated with limited lumbar discectomy and Barricaid device implantation. The trial was prospectively registered at ClinicalTrials.gov (NCT03986580) before first patient enrollment. The protocol for this clinical trial was approved by local institutional review boards and the Western Institutional Review Board (Puyallup, WA, United States). All enrolled patients provided written informed consent before study participation. Patient eligibility criteria, surgical technique, device characteristics, and follow-up methodology including imaging and outcome reporting in this study were comparable to those in an FDA-regulated RCT with the Barricaid device [6].

Patients

Patients underwent preoperative axial and sagittal magnetic resonance imaging (MRI) of the lumbar spine, 4-view X-rays, and physical and neurological examinations. Eligible patients presented with lumbar disc herniation (L4-L5 or L5-S1), leg pain severity ≥40 (0-100 scale) and Oswestry Disability Index (ODI) score ≥40 (0-100 scale) despite at least six weeks of nonsurgical management, and a positive straight leg raise sign on physical examination. Patients who met all preoperative eligibility criteria received limited lumbar discectomy after which the final eligibility criterion regarding annular defect size was assessed.

Surgical procedure

Patients who met the preoperative screening criteria were treated with limited lumbar microdiscectomy using an interlaminar transflaval approach as described by Spengler [7]. At completion of the discectomy, patients with a large annular defect (4-6 mm tall and 6-10 mm wide) received the Barricaid device, a permanent implant with a flexible woven polymer fabric component intended to occlude the annular defect and a bone anchor to secure the device to an adjacent vertebral body. Surgeons were instructed to perform discectomies in the usual fashion without enlarging the defect size for the purpose of inclusion into the study. In patients with smaller defects, the discectomy was completed in the usual fashion without device implantation and the patient was excluded from further trial participation.

Outcomes

Patient follow-up in the study occurred at four weeks and three months; patients will remain in follow-up through one year post-treatment. Anteroposterior and lateral X-rays were performed at each follow-up examination. Axial and sagittal MRI of the lumbar spine with or without contrast was performed at the three-month follow-up visit. Patients were also evaluated for leg pain severity, ODI score, adverse events, and occurrences of symptomatic reherniation and reoperation. The minimal clinically important difference (MCID) was defined as at least a 20-point decrease from baseline for leg pain [8], and at least a 15-point decrease from baseline for ODI [9]. Adverse events were adjudicated for seriousness and relation to the procedure or device by an independent data safety monitoring board. Symptomatic reherniation was defined as either confirmed reherniation during a reoperation; imaging core laboratory confirmation of reherniation based on MRI performed at an unscheduled visit due to patient symptoms; or imaging core laboratory confirmation of reherniation based on MRI performed at a scheduled visit in patients with ODI ≥40 and positive leg raise sign, or with an adverse event deemed related to reherniation, lumbar/leg pain, or a neurological event [10]. Index-level reoperation was defined as any surgical procedure performed at the level of the original herniation, regardless of side or reason. An independent imaging core laboratory read imaging studies to assess device status and anatomical characteristics following the procedure. 

Statistical analysis

Baseline characteristics were summarized with standard descriptive statistics. Categorical variables were described with percents and counts. Continuous variables were described with means and standard deviations. Longitudinal changes in leg pain severity and ODI score were evaluated with repeated measures analysis of variance and a p-value less than 0.05 represented a statistically significant change from baseline. All analyses were performed using STATA version 16.1 (StataCorp, College Station, TX, United States). 

## Results

Postmarket study results

A total of 55 patients were enrolled at 12 sites in the US between May 2020 and February 2021. Mean patient age was 41±13 years, 60% were male, and mean clinical symptom severity scores at baseline were 83±14 for leg pain and 58±15 for ODI. Imaging revealed disc herniation at L4/L5 (49%) or L5/S1 (51%) and intraoperative annular defect width measurements ranged from 6 to 10 mm (Table 1). The mean volume of nucleus removed during the limited discectomy procedures was 1.1±0.6 cc. 

**Table 1:** Baseline patient characteristics. Values are mean ± standard deviation (minimum, maximum) for continuous variables and % (n / N) for categorical variables.

Characteristic	Value
Age (years)	41 ± 13 (18, 75)
Male sex	60% (33/55)
Body mass index (kg/m^2^)	29 ± 4 (18, 37)
Smoking history	44% (24/55)
Level of herniation	
L4/L5	49% (27/55)
L5/S1	51% (28/55)
Herniation type	
Contained	56% (31/55)
Extruded	42% (23/55)
Sequestered	2% (1/55)
Annular defect width (mm)	
6	7% (4/55)
7	20% (11/55)
8	22% (12/55)
9	22% (12/55)
10	29% (16/55)
Leg pain severity	83 ± 14 (49, 100)
Oswestry Disability Index	58 ± 15 (40, 94)

**Figure 1:**
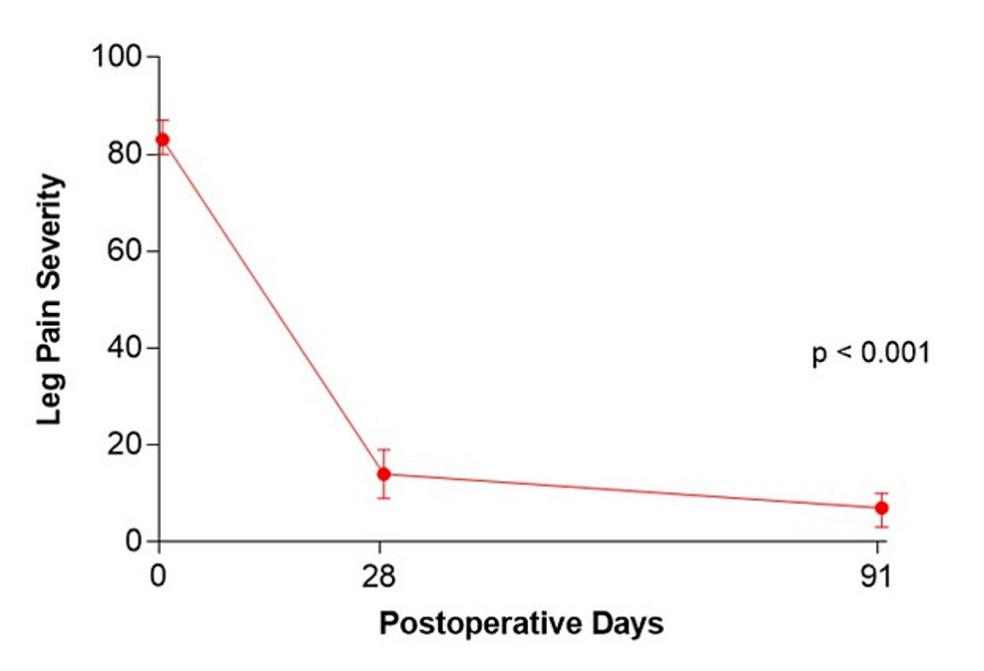
Change in leg pain severity over three months following lumbar discectomy and Barricaid device implantation.

**Figure 2:**
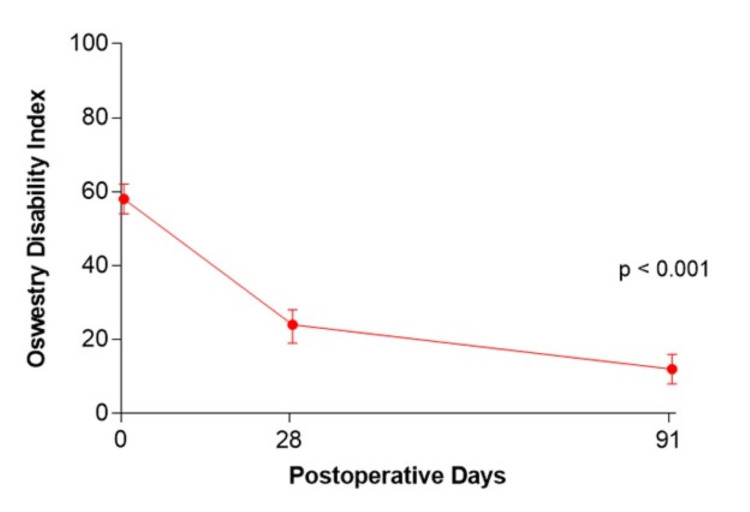
Change in Oswestry Disability Index over three months following lumbar discectomy and Barricaid device implantation.

Mean percent reduction in leg pain severity from baseline to three months was 92% (83 to 7) and the magnitude of symptom improvement met or exceeded the MCID in 98% (54/55) of patients (Figure 1). The mean percent reduction in ODI score was 79% (58 to 12) and the MCID was met or exceeded in 95% (52/55) of patients (Figure 2). Two (3.6%) patients experienced a symptomatic reherniation and one (1.8%) patient underwent a reoperation within three months. The first reherniation occurred two days postoperatively and a reoperation was performed. The second reherniation occurred on day 92 which was managed nonoperatively. Four serious adverse events were reported in three (5.5%) patients, which included disc herniation (previously mentioned), neurological function decline, infection, and hematoma. No device migrations or fractures were observed.

Comparison of Barricaid device performance in postmarket study vs. FDA-regulated RCT

A secondary purpose of the study was to descriptively compare these early real-world outcomes to those from an FDA-regulated RCT [6]. Baseline patient characteristics were comparable in the postmarket study vs. the FDA RCT, including age (41 vs. 43 years), male sex preponderance (60% vs. 57%), affected lumbar level (primarily L4/L5 or L5/S1), annular defect width (6-10 mm range), and clinical symptom severity. Over three months of follow-up, symptom improvement and the rates of symptomatic reherniation (3.6% vs. 2.3%) and reoperation (1.8% vs. 1.9%) were comparable in the studies (Table 2).

**Table 2:** Descriptive comparison of Barricaid device results through three months in postmarket study vs. FDA-regulated RCT. ODI, Oswestry Disability Index; RCT, randomized controlled trial.

Characteristic	Postmarket Study (n=55)	FDA-regulated RCT (n=272)
Baseline patient characteristics		
Age, yr	41±13	43±11
Male sex, %	60%	57%
Body mass index, kg/m^2^	29±4	26±4
Index level, %		
L2-L3	0%	1%
L3-L4	0%	3%
L4-L5	49%	45%
L5-S1	51%	51%
Leg pain severity	83±14	81±15
ODI	58±15	59±12
Outcomes through 3 months follow-up		
Mean percent reduction in leg pain severity	92%	87%
Mean percent reduction in ODI	79%	74%
Symptomatic reherniation	3.6%	2.3%
Reoperation	1.8%	1.9%

## Discussion

Annular healing following lumbar discectomy yields biomechanically inferior fibrous tissue with reduced capacity to accommodate tensile force [11-13]. This makes reherniation of nuclear material more likely to occur in patients with large post-surgical annular defects. The goals of this study were to determine early clinical outcomes with the Barricaid device in real-world conditions and to descriptively compare outcomes to those from an FDA-regulated RCT using the same device [6]. Ultimately, the early results from this postmarket study demonstrated clinically meaningful improvements in clinical symptoms and low rates of symptomatic reherniation and reoperation, which were comparable to those in the strictly controlled FDA-regulated RCT.

An interesting aspect of this paper was the relatively short duration for reporting of results. Although patients will continue to be followed for one year, three-month outcomes in this patient population are predictive of longer term results [10]. These study results also favorably compare to the 7.6% symptomatic reherniation rate observed over three months of follow-up in patients with large annular defects treated with discectomy without the device [6]. Additionally, most reherniations occur within the first year which provides clinical justification for the one-year follow-up duration [14]. Considering that most symptomatic reherniations occur early in follow-up [14], that 80% of patients with symptomatic reherniation require reoperation [5], and that reoperations are more technically demanding and expensive than primary procedures [15], there is potential for the Barricaid device to provide early cost savings in this high-risk patient population.

There were several limitations of this study. First, this report was focused on results during the first three months following surgery which has been identified as a critical period whereby the risk of herniation recurrence is high. Of course, a complete one-year follow-up in the trial will be required to confirm these favorable early results. Second, Barricaid device implantation is not appropriate for all lumbar discectomy cases. Approximately one in three patients have a large annular defect after lumbar discectomy [5] and these patients have a high risk for reherniation. Therefore, device implantation is only appropriate in this subset of patients. Annular closure in patients with small annular defects is not clinically indicated. Finally, this study was not designed to statistically demonstrate noninferiority of outcomes comparing Barricaid device utilization in the postmarket study vs. the FDA-regulated RCT since such a design would require enrollment of more than 1,000 patients. However, considering that early clinical results between these populations as well as patient eligibility criteria, surgical technique, device characteristics, and follow-up methodology were comparable, this trial provides reasonable evidence that the Barricaid device performs consistently in real-world use as well as in strictly controlled clinical trials.

## Conclusions

Among patients with large annular defects following lumbar discectomy treated with the Barricaid device in real-world conditions, early results demonstrated clinically meaningful improvements in patient symptoms and low rates of symptomatic reherniation, reoperation, and complications. These results were comparable to those observed with the Barricaid device in an FDA-regulated trial. Reherniation rates after lumbar discectomy with Barricaid device implantation are comparably low in clinical trial settings and in real-world use.
